# Immersed in a reservoir of potential: amniotic fluid-derived extracellular vesicles

**DOI:** 10.1186/s12967-024-05154-2

**Published:** 2024-04-12

**Authors:** Ishara Atukorala, Natalie Hannan, Lisa Hui

**Affiliations:** 1 Department of Obstetrics, Gynaecology & Newborn Health, Melbourne Medical School, The University of Melbourne, Mercy Hospital for Women, 163 Studley Road, Heidelberg, VIC 3084 Australia; 2 Department of Obstetrics, Gynaecology & Newborn Health, The Northern Centre for Health Education and Research, Northern Health, Epping, VIC Australia; 3 Department of Perinatal Medicine, Mercy Hospital for Women, Mercy Health, Heidelberg, VIC Australia; 4 Reproductive Epidemiology Group, Murdoch Children’s Research Institute, Parkville, VIC Australia

**Keywords:** Extracellular vesicles, Exosomes, Amniotic fluid, Amniotic fluid stem cells, Pregnancy, Biomarkers, Therapy

## Abstract

This review aims to encapsulate the current knowledge in extracellular vesicles extracted from amniotic fluid and amniotic fluid derived stem/stromal cells. Amniotic fluid (AF) bathes the developing fetus, providing nutrients and protection from biological and mechanical dangers. In addition to containing a myriad of proteins, immunoglobulins and growth factors, AF is a rich source of extracellular vesicles (EVs). These vesicles originate from cells in the fetoplacental unit. They are biological messengers carrying an active cargo enveloped within the lipid bilayer. EVs in reproduction are known to play key roles in all stages of pregnancy, starting from fertilisation through to parturition. The intriguing biology of AF-derived EVs (AF-EVs) in pregnancy and their untapped potential as biomarkers is currently gaining attention. EV studies in numerous animal and human disease models have raised expectations of their utility as therapeutics. Amniotic fluid stem cell and mesenchymal stromal cell-derived EVs (AFSC-EVs) provide an established supply of laboratory-made EVs. This cell-free mode of therapy is popular as an alternative to stem cell therapy, revealing similar, if not better therapeutic outcomes. Research has demonstrated the successful application of AF-EVs and AFSC-EVs in therapy, harnessing their anti-inflammatory, angiogenic and regenerative properties. This review provides an overview of such studies and discusses concerns in this emerging field of research.

**Supplementary Information:**

The online version contains supplementary material available at 10.1186/s12967-024-05154-2.

## Introduction and background

### Composition of amniotic fluid

Amniotic fluid (AF) is a unique conditioning medium for the developing fetus throughout gestation until birth [1]. The composition and volume of AF changes across gestation and aligns with key gestational stages [2]. The AF volume increases linearly from first trimester until about 33 weeks gestation and then reduces towards full-term [3]. It starts as a by-product of maternal serum consisting of water and electrolytes and gradually changes to fetal products by the late second trimester [1, 4–6]. In the early weeks of gestation, the fetal skin is a simple epithelium layer, as such AF freely diffuses across [5]. However, after keratinization completes, around week 25, fetal urination becomes the main source of increasing AF volume, while fetal lung secretions also contribute significantly [3]. Fetal “respiration” and swallowing remain the principal routes for AF resorption [3, 7]. At term, the human fetus produces 800–1200 ml of urine per day, which can replace the entire AF volume within 12–24 h [8, 9].

AF is rich in numerous nutrients and growth factors supporting fetal development [10], while antibodies and antibacterial agents present within the fluid help to protect the fetus from infections [11]. Apart from playing an integral part in fetal health, AF has been a useful prenatal diagnostic sample, since amniocentesis was first performed in the late 1960s for fetal karyotyping [1].

### What are extracellular vesicles?

Extracellular vesicles (EVs) are lipid-bilayer membrane-enclosed vesicles that are secreted by virtually all cells [12]. Their diameter can range from small EVs of 30–150 nm to oncosomes of 10 µm [13]. Since the first description of EVs in the 1980s [14, 15], EVs have been extensively researched in health and disease. There are many classes of EVs, including exosomes, oncosomes, shedding microvesicles, migrasomes and apoptotic bodies. The categorisation is based on their biogenesis and secretion mechanisms, size, and function [16–18]. EVs secreted by the host cells can mediate both proximal and distal signalling events in organisms [19–21]. Their biological cargo is transported intact, avoiding degradation through the protection of the lipid bilayer membrane [22]. Their unrestrictive crossing of the blood–brain barrier makes them an appealing delivery mode for central nervous system therapeutics [23, 24].

### EVs as a method of studying human reproduction

EVs have been a valuable source of information about human reproduction. Examples include uterine luminal fluid EVs in fertilisation, maintaining the sperm viability in the oviduct and continuity of pregnancy by keeping Ca^2+^ homeostasis [25]. The potential influence can be attributed to their selectively packaged cargo [26]. They appear to play a critical role in embryo implantation, establishing the first communication between the mother and the conceptus [27, 28]. Placental EVs are known to influence uterine spiral arterial remodelling under physiological conditions, but might be compromised under pathological conditions [29].The role of AF-EVs in parturition [30, 31] is discussed later in detail.

It is evident that the molecular signature of AF-EV cargo changes according to feto-maternal pathologies, creating opportunities for many clinical applications. Pregnancy complications such as pre-eclampsia [32] and preterm labour [30, 33], fetal complications such as congenital hydronephrosis [34] and fetal alcohol syndrome [35] have been studied using AF-EV borne molecules, which are discussed later in detail. While these studies are beneficial in biomarker discovery and knowledge gain, they are yet to achieve clinical translation.

### Amniotic fluid EVs and amniotic fluid stem/stromal cell EVs in therapy

Therapeutic applications of EVs have been investigated by researchers, mostly as drug delivery vehicles [23, 24, 36]. However, AF-EVs and AFSC-EVs are more than a transport mode for exogenous therapeutics. They are loaded with endogenous molecules with therapeutic potential, that can influence tissue regeneration, anti-inflammation, paracrine signalling, and immunomodulation [37, 38]. Unmodified EVs isolated from term AF have been tested in pre-clinical models to treat conditions such as bronchopulmonary dysplasia [39] and azoospermia [40]. They have also been used in human trials to treat severely ill COVID-19 patients. Case studies performed in the USA demonstrated the safe clinical use of AF-EVs in humans, successfully improving lung function of intubated COVID-19 patients [41, 42].

EVs derived from amniotic fluid stem cells/stromal cells (AFSC-EVs) are a popular choice for therapeutic experimentation in pre-clinical models, owing to the easy access to the source material and successful laboratory production. The studies included in this review used several distinct terms to identify the cell populations—stem cells, mesenchymal stem cells and mesenchymal stromal cells*.* The field of stem cell research acknowledges the potential ambiguity in cell nomenclature by various research groups [43–45]. Therefore, for the purpose of this review, we have used AFSC-EVs to identify EVs derived from the conditioned media of all three different cell types mentioned.

EVs from AF stem cell cultures appear to have a more consistent paracrine profile than stem cells, thus avoiding the unpredictability that is tied with stem cell therapy [38]. AFSC-EVs have produced positive responses in preclinical studies of various pathologies, including premature ovarian failure [46], cardiac injury [47, 48], neuroinflammation [49, 50] and necrotising enterocolitis [51, 52].

The aim of this narrative review is to summarise the current knowledge of AF-EVs and AFSC-EVs, including their isolation and characterisation, physiological and pathological implications, and potential clinical applications. Due to the variability in methods used to isolate EVs, studies discussed in this review include a wide range of EV sizes and categories with varying molecular properties, including microparticles, microvesicles, exosomes and nanovesicles (Table 1).

**Table 1:** Vesicle types included in this review

Vesicle type	Description (as indicated in the studies)	Vesicle size range (nm)
Exosomes	Includes both ultracentrifugation-based crude extractions and further purified vesicles using density gradient centrifugation, filtration, or chromatographic methods	30–150
Microparticles	Isolated using a 13,000–18,000*g* centrifugations	100–200
Microvesicles	Isolated using a final 100,000*g* ultracentrifugation	100–400
Nanovesicles	Isolated using a final 100,000*g* ultracentrifugation	40–200
Extracellular vesicles	Isolation methods vary: 20,000–200,000*g* centrifugations, commercial kits and polymeric precipitation methods May or may not involve further purification using density gradient centrifugation, filtration, or chromatographic methods	30–1000
Small extracellular vesicles	Isolated using a 100,000*g* ultracentrifugation	30–150

This review discusses several types of vesicles as named in the research studies included. Irrespective of similar isolation methods and overlapping vesicle sizes, some EV populations were named differently, or vice-versa

## Selection of studies

PubMed Central was searched on the 13th of June 2023, using the keyword combination (exosomes OR extracellular vesicles) AND amniotic fluid, using the advanced search option. A total of 148 search results published from 2000 to June 2023 was retrieved. Articles were included if they were full manuscripts published in English reporting original research on EVs directly isolated from AF or from AF stem cell cultures.

**Fig. 1:**
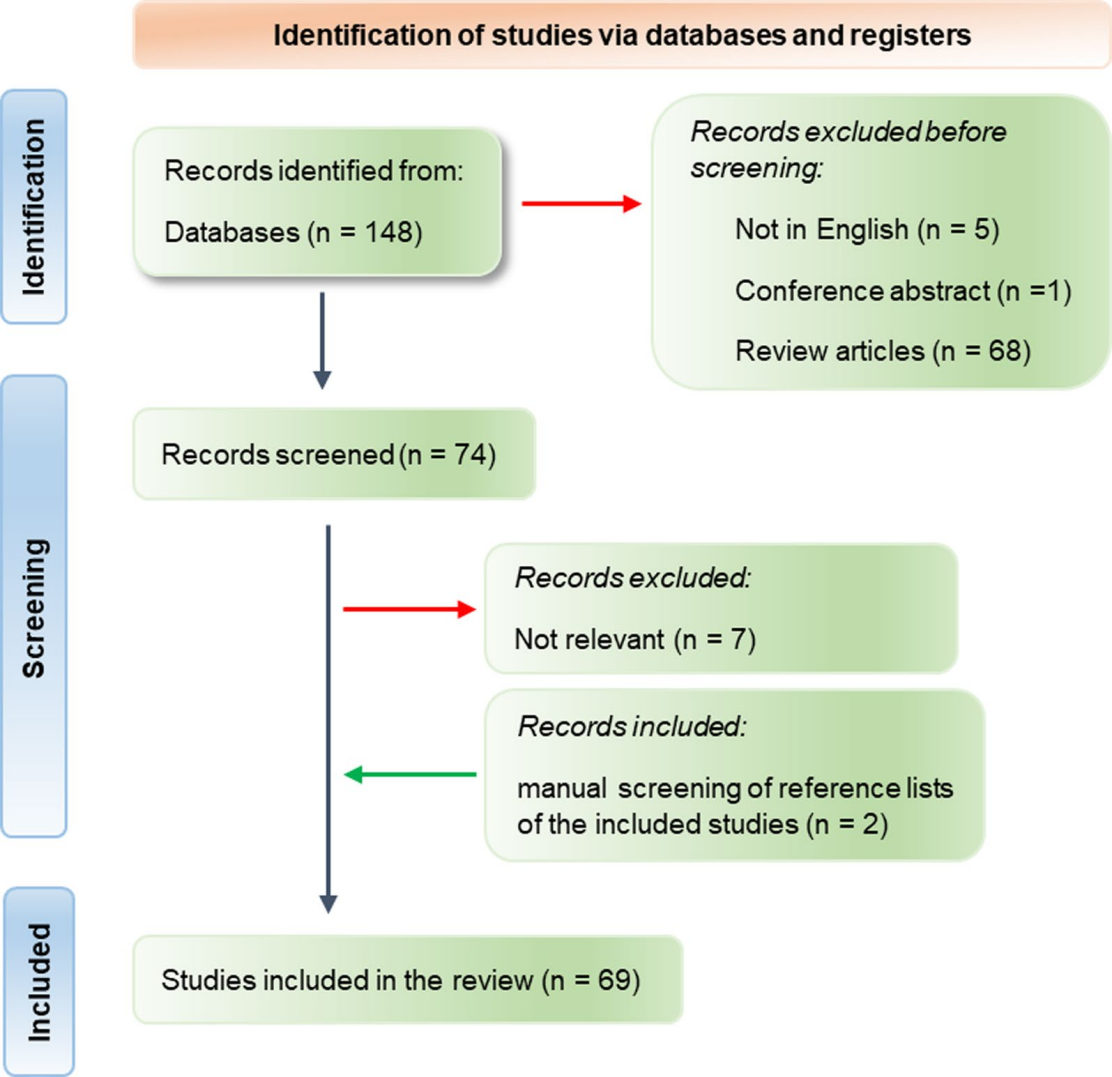
PRISMA flow chart of the study selection criteria for the review. A thorough literature search via NCBI Pubmed resulted in 148 articles, of which 69 were included in this review, after excluding irrelevant studies

A list of 74 articles was selected for full-text review after screening of titles, abstracts, and keywords, of which 7 irrelevant studies were excluded. Two articles were retrieved after a manual search of reference lists of included articles. A total of 69 full-text articles were included (Additional file 1. List of included studies) (Fig. 1). Forty-four (64%) studies were published since 2020. We performed a narrative overview and content synthesis of the final included articles.

## AF-EV isolation

### The source of AF

The majority of studies derived human AF samples from clinically-indicated amniocentesis (18), term labour or Caesarean section (13). Three studies did not state the source of AF. Two other groups studied murine and ovine AF (Table 2).

**Table 2:** Isolation methods to obtain EVs from amniotic fluid

Isolated EV population	Sample source	Initial centrifugation steps Spin (g), time (min)	EV isolation method	Further purification
Exosomes [22]	Amniocentesis (approximately 16 weeks)	i) 300*g*, 20 min ii) 10,000*g*, 20 min	100,000*g*, duration not clear	Sucrose density gradient centrifugation at 100,000*g*, 2.5 h
Exosomes [30]	Collection at labour/caesarean section	i) 2000*g*, 30 min ii) 2000*g* 45 min	100,000*g*, 2 h	i) Filtration through a 0.22 μm filter ii) Centrifugation at 100,000*g*, 2 h
Exosomes [31]	Collection at labour/caesarean section	i) 300*g*, 10 min ii) 2000*g*, 30 min iii) 12,000*g*, 45 min	Filtration through a 0.22-μm filter and centrifugation at 120,000*g*, 70 min, twice	
EVs [32]	Caesarean section	i) 300*g*, 5 min ii) 500*g*, 10 min iii) 10,000*g*, 30 min in a cell sieve	100,000*g*, 2 h	i) Filtration through a 0.22 μm filter ii) Size-exclusion chromatography
EVs [33]	Amniocentesis (17–36 weeks)		Commercial EV isolation Kit	
Exosomes [34]	Amniocentesis	i) 300*g*, 20 min ii) 10,000*g*, 20 min	100,000*g*, 2.5 h	Sucrose density centrifugation at 100,000*g*, 2.5 h
Exosomes (murine) [35]	Collection with a needle directed to amniotic cavity after euthanization	i) 300*g*, 20 min ii) 10,000*g*, 30 min	120,000*g*, > 18 h	
EVs [39]	Caesarean section	Filtration system (details unavailable)	100 000*g*, 3 h	
Exosomes (sheep) [40]	Not specified	i) 300*g*, 10 min ii) 2000*g*, 15 min iii) 10,000*g*, 30 min iv) Filtration through 0.22 μm-sized microfilter	100,000*g*, 1 h	
EVs (commercial product: Zofin) [41, 42, 122]	Caesarean section		Centrifugation and filtration	
EVs [53]	Not specified	i) 3000*g*, 20 min ii) 20,000*g*, 20 min	Incubate with DTT 20,000*g*, 20 min	
Exosomes [54]	Collection at labour/caesarean section	i) 300*g*, 10 min ii) 2000*g*, 20 min iii) 10,000*g*, 30 min	100,000*g*, 2 h	Commercial EV isolation Kit
Exosomes [55]	Amniocentesis (15–16 weeks)	i) 48,298*g*, 30 min ii) Filtration through 0.22 µm filter	584,401*g*, 60 min	
Microparticles [67]	Collection at labour/caesarean section	1500*g*, 15 min	13,000*g*, 2 min	
Exosomes [68]	Amniocentesis (approximately 16 weeks)	i) 300*g*, 10 min ii) 10,000*g*, 20 min	i) 100,000*g*, 2 or 18 h for human AF-EVs ii) 120,000*g*, overnight for murine AF-EVs	
Exosomes [70]	Amniocentesis	i) 300*g*, 5 min ii) 1200*g*, 20 min iii) 10,000*g*, 30 min	100,000*g*, 1 h	Sucrose density gradient centrifugation (1.08–1.24*g* sucrose/ml) at 150,000*g*, 12 h
EVs [72]	Caesarean section	i) 500*g*, 10 min ii) 2000*g*, 15 min iii) Filtered through a 0.22 μm	100,000*g*, 3 h	
EVs [73]	Amniocentesis (16–17 weeks)	i) 300*g*, 10 min ii) 2000*g*, 20 min iii) 10,000*g*, 45 min	100,000*g*, 1 h	
EVs [74]	Caesarean section		Commercial EV isolation Kit or 107,000*g*, 1.5 h	
Exosomes [81]	Amniocentesis	i) 300*g*, 5 min ii) 1200*g*, 20 min iii) 10,000*g*, 30 min	100,000*g*, 1 h	
EVs [84]	Amniocentesis	i) 1000*g*, 15 min ii) 2000*g*, 15 min iii) 3000*g*, 15 min	110,000*g*, 75 min	
Exosomes [85]	Amniocentesis (19–23)	i) 300*g*, 10 min ii) 2000*g*, 30 min iii) 12,000*g*, 45 min	110,000*g*, 2 h	i) Filtration through a 0.22-μm filter ii) Centrifugation at 110,000*g*, 70 min
Exosomes [86]	Amniocentesis (15–25 weeks)		Commercial EV isolation Kit	
Exosomes [92]	Amniocentesis (18–20 weeks)	Unclear	100,000*g*, duration unclear	
EVs [93]	Amniocentesis (16–20 weeks)	400*g*, 10 min	EVs in the AF were stained (not isolated)	
Microparticles [94]	Collection at Caesarean section	1500*g*, 15 min	18,000 rpm, 30 min	
EVs [95]	Amniocentesis (15–18 weeks)	i) 250*g*, 5 min ii) Filtration through a 0.1 µm pore membrane	20,000*g*, 30 min	
EVs [96]	Amniocentesis (15–28 weeks)		Size exclusion chromatography	
EVs [98]	Amniocentesis (approximately 17 weeks)		Commercial EV isolation Kit	
EVs [69, 123]	Amniocentesis (16–18 weeks)	i) 300*g*, 10 min ii) 3000*g*, 20 min iii) 17,000*g*, 25 min	100,000*g*, 2 h	Sucrose density gradient centrifugation at 100,000*g*, 2 h OR ion-exchange chromatography
Exosomes [124]	Caesarean section	i) 300*g*, 10 min ii) 2000*g*, 20 min iii) 10,000*g*, 30 min	100,000*g*, 1 h	
EVs [125]	Not specified	i) 3000*g*, 15 min ii) 11,000*g*, 15 min iii) 14,000*g*, 15 min iv) Filtered through a 0.22 μm	100,000*g*, 1 h	

Different AF-EV isolation methods were observed even within the same research group, presumably due to changed consistencies in patient samples. Gestation (in weeks) is mentioned where possible for amniocentesis samples. *min* minutes, *h *hours

### Lack of standardization in AF-EVs isolation methods

The most common method to isolate small AF-EVs was differential centrifugation coupled with ultracentrifugation. The majority of studies performed centrifugation at 300*g* for 15 min to remove cells, followed by 2000*g* for 20 min to eliminate cellular debris. This step was most commonly followed by centrifugation at 10,000*g* for 30 min and filtration to remove larger vesicles. Ultracentrifugation at 100,000–120,000*g* for varying time periods pelleted down small EVs.

Various methods were reported for further purification of EVs following ultracentrifugation. While some researchers opted for density gradient centrifugation or ion exchange chromatography, others used commercially available kits for EV isolation (Table 2). Researchers preferred amniocentesis for sample collection over Caesarean section and differential centrifugation for EV isolation as indicated in Table 3 (a summary of Table 2).

**Table 3:** Summary of Table 2

	Number of studies
AF collected from amniocentesis	18
AF collected from term labour/caesarean section	13
AF collected from pre-clinical models	2
Used commercial EV isolation kits to isolate EVs	5
Used centrifugation methods to isolate EVs	32
Used filtration to separate larger vesicles prior to isolating smaller vesicles	8
Used centrifugation (~ 10,000*g* spin) to separate larger vesicles prior to isolating smaller vesicles	13
Used both centrifugation (~ 10,000*g* spin) and filtration in tandem to separate larger vesicles prior to isolating smaller vesicles	2
Used further purification methods to clean the isolated EVs	8

This summary of Table 2 provides a study count according to the type of starting samples, EV isolation methods used in each study

Ebert and Rai developed an unconventional three-step centrifugation protocol to isolate AF-EVs, that involved addition of dithiothreitol (DTT) to the EV pellet to denature external protein aggregates [53]. This method may not be suitable for studies focusing on EV membrane proteins as DTT can denature the ectodomains of proteins. Others used a centrifugation-based method in combination with filtration and commercially available chromatography columns for EVs isolation from small volumes (down to 250 µL) of AF [54]. A comparison of methods study stated that ultracentrifugation resulted in better EV yield from human AF than commercial exosome isolation reagents [55].

The variability in methods may partly be due to the variability in samples. For example, term AF contains vernix caseosa (white wax-like substance covering the fetal skin) compared to second trimester AF, requiring strenuous sample cleaning steps. While AF can be a challenging sample, one would expect to have largely consistent methods for EV isolation from conditioned media derived from cell cultures.

## Amniotic fluid stem/stromal cell EV isolation

### Amniotic fluid stem/stromal cell cultures are used as a reliable supply of EVs

Many researchers have isolated AF stem or stromal cells and cultured them to provide a convenient and continuous in vitro source of EVs. These studies used human/murine primary or cryopreserved cells obtained from second-trimester amniocentesis, elective Caesarean sections or both. Five research groups obtained mouse AF stem cells (Table 4), presumably to maintain the consistency with experimental animal models. Table 5 summarises this information, providing a count of studies that used different sample sources and EV isolation methods.

**Table 4:** Isolation methods to obtain EVs from conditioned culture media of AF stem/stromal cells

Isolated EV population (as mentioned in the study)	Source of cells	Cell culture media for EV isolation	Culture conditions	Culture time for EV isolation (hours)	Initial spins/preparations	EV isolating spin/method
Exosomes [46]	Aspiration through amniotic sac (primary murine AFSC)	MEM-α	37 °C, 5% CO_2_	48	i) 2000*g*, 20 min ii) Filtration through 0.22 μm filter	Commercial EV isolation Kit
EVs [47]	Amniocentesis (primary human AFSC)	Sterile phosphate-buffered saline	37 °C, 5% CO_2_	24	i) 300*g*, 5 min ii) 2000*g*, 20 min	200,000*g*, duration not specified
EVs [48]	Amniocentesis (primary human AFSC)	αMEM with 15% FBS, 1% l-glutamine, 1% penicillin/streptomycin, 18% Chang B and 2% Chang C	37 °C, 5% CO_2_, 1% O_2_ and (hypoxia)	24	i) 3000*g*, 20 min ii) 10,000*g*, 15 min	100,000*g*, 70 min
Exosomes [49]	Not specified (primary human AFSC)	IDMEM with exosome-free FBS, 100 μg/mL streptomycin/penicillin, 2 mM l-glutamine, 5 ng/mL basic fibroblast growth factor 2	37 °C, 5% CO_2_	72	i) 200*g*, 5 min ii) 200*g*, 10 min iii) 16,500*g*, 30 min	120,000*g*, 90 min
EVs [50, 57]	Amniocentesis (primary human AFSC)	FBS free α-MEM with 2 mM l-glutamine, 100 U/ml penicillin/streptomycin	Not specified	96	Concentrating using centrifugal filter unit with 3 K cut-off	Commercial EV isolation Kit
Exosomes [51]	Not specified (cryopreserved murine AFSC)	Serum free MEM-α with 1% penicillin/streptomycin/amphotericin B	Not specified	48	i) 300*g*, 10 min ii) 2000*g*, 10 min iii) 10,000*g*, 30 min	100,000*g*, 70 min
EVs [52]	Aspiration through amniotic sac (primary murine AFSC)	Serum free α-MEM with 20% Chang medium C, 1% penicillin/streptomycin	37 °C, 5% CO_2_	24		Commercial EV isolation Kit
Exosomes [56]	Amniocentesis (primary human AFSC)	FBS free α-MEM with 2 mM l-glutamine, 100 units/mL penicillin/streptomycin	37 °C, 5% CO_2_	96	Concentration using a 3 KDa cut-off filter	Commercial EV isolation Kit
Exosomes [58]	Amniocentesis (primary human AFSC)	FBS free α-MEM with 2 mM l-glutamine, 100 U/mL penicillin/streptomycin	37 °C, 5% CO_2_	96	i) 300*g*, 10 min ii) 10,000*g*, 30 min	100,000*g*, 1.5 h
Exosomes [59]	Amniocentesis (primary human AFSC)	DMEM: F12 (1:1) with 10 ng/mL bFGF, 10 ng/mL EGF, 10% fetal bovine serum, and 2 mM l-glutamine	37 °C, 5% CO_2_	Not specified	i) 500*g*, 10 min ii) 16,500*g*, 20 min iii) 0.22 μm filtration	118,000*g*, 70 min
Small EVs [60]	Not specified (primary human AFSC)	Serum free DMEM with 25 mM glucose, 4 mM GlutaMAX, 50 units/mL penicillin/streptomycin	37 °C, 5% CO_2_	24 or 48	i) 300*g*, 10 min ii) 10,000*g*, 40 min iii) Concentration using a 30 kDa filter	Commercial EV isolation Kit
EVs [61, 77]	Amniocentesis (primary human AFSC)	Serum free MSCBM with l-glutamine, gentamicin sulphate/amphotericin B	Not specified	Not specified	i) 300*g*, 10 min ii) 2000*g*, 20 min iii) 10,000*g*, 30 min	100,000*g*, 1 h
EVs [62]	Amniocentesis (primary human AFSC)	DMEM with 10% exosome depleted FBS	Not specified	72	*Ultracentrifugation*: 10,000*g*, 45 min *PEG*: Conditioned media mixed 1:1 with PEG 2000, 12 h, 4 °C	*Ultracentrifugation*: 100,000*g*, 70 min *PEG:* 10,000*g*, 20 min
EVs [64, 82]	Amniocentesis and caesarean section (primary human AFSC)	Serum free DMEM with 1% l-glutamine and 1% penicillin/streptomycin	37 °C, 5% CO_2_, 20% O_2_ (normoxia) or 37 °C, 5% CO_2_, 1% O_2_ and _(_hypoxia)	24	i) 300*g*, 10 min ii) 2000*g*, 20 min iii) Concentrating using a 3 kDa cut-off filter iv) 10,000*g*, 40 min	i) 100,000*g*, 2 h ii) Filtration through a 0.22 µm pore filter
EVs [65, 117]	Amniocentesis (primary human AFSC)	α-MEM with 7.5% exosome, depleted FBS	37 °C, 5% CO_2_	18	i) 300*g*, 10 min ii) 1200*g*, 10 min	100,000*g*, 14 h
Nanovesicles [75]	Amniocentesis (primary human AFSC)	Serum free MSCBM medium	Not specified	Not specified	i) 300*g*, 10 min ii) 2000*g*, 20 min iii) 10,000*g*, 30 min	100,000*g*, 1 h
Exosomes [76]	Amniocentesis (cryopreserved human AFSC)	Serum free DMEM with penicillin/streptomycin	37 °C, 5% CO_2_	24	2000*g*, 10 min	High speed, 1 h
EVs [80]	Amniocentesis (cryopreserved human AFSC)	DMEM with 10% EV depleted FBS, 1% glutamine, 1% antibiotics	37 °C, 5% CO_2_	Not specified	i) 800*g*, 30 min ii) 16,000*g*, 45 min	i) 100,000*g*, 2 h ii) Sucrose gradient centrifugation 100,000*g*, 16 h
EVs [99, 116]	Aspiration through amniotic sac (primary murine AFSC)	α-MEM with 7.5% exosome-depleted FBS	37 °C, 5% CO_2_	18	i) 300*g*, 10 min ii) 1200*g*, 10 min	100,000*g*, 14 h
Exosomes [102]	Amniocentesis (primary human AFSC)	DMEM with 0.5% exosome-depleted FBS	37 °C, 5% CO_2_	48	i) 1000*g*, 5 min ii) 3225*g*, 15 min iii) Concentrating using a 3 KDa cut-off filter	37,500 rpm, 2 h
Exosomes [104]	Amniocentesis (primary human AFSC)	10% exosome free FBS DMEM	37 °C, 5% CO_2_	48	i) 2000*g*, 10 min ii) 10,000*g*, 30 min	100,000*g*, 70 min
Exosomes [105]	Amniocentesis (primary human AFSC)	Not specified	37 °C, 5% CO_2_	Not specified	i) 300*g*, time not specified ii) 1000*g*, 5 min iii) 10,000*g*, 10 min iv) 0.1 μm filtration	100,000*g*, 1 h
Microvesicles [115]	Not specified (cryopreserved human AFSC)	DMEM with 15–20% FBS, 1% penicillin/streptomycin	Not specified	Not specified	300*g*, 5 min	100,000*g*, 2 h
EVs [126]	Amniocentesis (primary human AFSC)	Serum free MEM-α with 1% l-glutamine and 1% penicillin/streptomycin	37 °C, 5% CO_2_, 20% O_2_ (normoxic) or 37 °C, 5% CO_2_, 1% O_2_ and _(_hypoxic)	24	i) 3000*g*, 20 min ii) 10,000*g*, 15 min	100,000*g*, 70 min
EVs [127]	Aspiration through amniotic sac (cryopreserved murine AFSC)	Serum free RPMI-1640	Not specified	Overnight	6000*g*, 20 min	100,000*g*, 2 h
EVs [128]	Aspiration through amniotic sac (primary murine AFSC)	Serum free DMEM minimum essential medium with 100 IU/mL penicillin/streptomycin	37 °C, 5% CO_2_	48	i) 300*g*, 5 min ii) 16,500*g*, 40 min iii) Filter through 0.22 μm	120,000*g*, 70 min
EVs [100]	Not specified (cryopreserved human AFSC)	FBS free α-MEM	37 °C, 5% CO_2_	24	i) 180*g*, 5 min ii) 1000*g*, 10 min iii) 2000*g*, 10 min iv) Filter through 0.22 μm	100,000*g*, 16 h
Exosomes [129]	Caesarean section (primary human AFSC)	10% exosome free FBS supplemented media	37 °C, 5% CO_2_	48	i) 3000*g*, 15 min ii) 13,000*g*, 30 min	100,000*g*, 60 min/commercial EV isolation Kit (unclear)
EVs [103]	Amniocentesis (primary human AFSC)	FBS free α-MEM with 2 mM l-glutamine, 100 U/mL penicillin/streptomycin	37 °C, 5% CO_2_	96	i) 300*g*, 10 min ii) 10,000*g*, 30 min	100,000*g*, 1.5 h
Exosomes [101]	Not specified (cryopreserved human AFSC)	CHANG Amnio (Irvine) culture medium supplemented with 10% exosome-depleted FBS and 1% penicillin/streptomycin	37 °C, 5% CO_2_	24–48	i) 750*g*, 5 min ii) 1500*g*, 5 min iii) 14,000*g*, 35 min	i) 110,000*g*, 2 h ii) 0.22 μm filtration

Scientists chose different sources of amniotic fluid derived cells and a variety of cell culture conditions. Cell culture period before EV isolation varied from 18–96 h

*α-MEM* minimum essential medium, *IDEMEM*: Iscove’s modified Dulbecco’s medium, *FBS* fetal bovine serum, *DMEM* Dulbecco’s Modified Eagle’s Medium

**Table 5:** Summary of Table 4

	Number of studies
Primary cells derived from AF collected at amniocentesis	18
Primsary cells derived from AF collected at term labour/caesarean section	3
Cryopreserved human cells	5
Cells derived from AF of pre-clinical models	7
Used commercial EV isolation kits to isolate EVs	7
Used centrifugation methods to isolate EVs	28
Used filtration to separate larger vesicles prior to isolating smaller vesicles	7
Used centrifugation (~ 10,000*g* spin) to separate larger vesicles prior to isolating smaller vesicles	13
Used both centrifugation (~ 10,000*g* spin) and filtration in tandem to separate larger vesicles prior to isolating smaller vesicles	4
Used further purification methods to clean the isolated EVs	1

This summary of Table 4 provides a study count according to the source of stem/stromal cells, EV isolation methods used in each study

Stem cells were most commonly isolated from AF by fluorescence activated cell sorting for c-Kit expression [47, 48, 52, 56–58] or for CD44/CD105 expression [59]. Other researchers cultured cells from AF and separated the colonies based on the fibroblast morphology of the cells [60, 61]. Whether these different methods impact EV biogenesis and secretion pathways differently in stem cells is yet to be understood.

Majority (79%) of the AFSC-EV studies included in this review referred to their cell populations as stem cells while 2 studies mentioned the isolation of mesenchymal stromal cells. Five other studies mentioned the use of mesenchymal stem cells. Table 4 describes different culture conditions used by research groups to grow the isolated cells.

### A variety of isolation methods for AF stem/stromal cell EVs

There is a variety of methods of EV isolation from AF stem cell-conditioned media, but most employed some form of differential centrifugation with many variations in the centrifugation steps. Studies published in the past 2–3 years commonly used the classic approach of differential centrifugation steps to remove live and dead cells (500*g*), cell debris (2000*g*), large vesicles (10,000–15,000*g*) and a final ultracentrifugation collecting small EVs (100,000–120,000*g*) (Table 4). A recent study comparing ultracentrifugation and a novel polyethylene glycol (PEG)-based EV precipitation method demonstrated that PEG-based isolation produced approximately five times more EV yield and EV proteins, but one third the EV-RNA content compared to ultracentrifugation [62]. The choice of isolation method may consequently influence the properties of EVs [62].

Isolation methods depend on the differential density, solubility factors and size of the target EVs [63]. Efforts to standardize EV research by the International Society for Extracellular Vesicles is reflected in the studies published since 2020, with a degree of consistency in methods compared to earlier studies. However, all methods result in some degree of variation in size range, purity and protein content of each EV preparation. Some research groups have attempted to standardize their laboratory protocols by adhering to good manufacturing practices (GMP) guidelines [41, 42, 64], or used GMP-grade AF stem cells for culture [65]. This is an essential step in ensuring that the findings from basic research can eventually be translated into clinical applications and scaled up into commercial products.

## Characterisation of EVs should adhere to internationally accepted guidelines

**Fig. 2:**
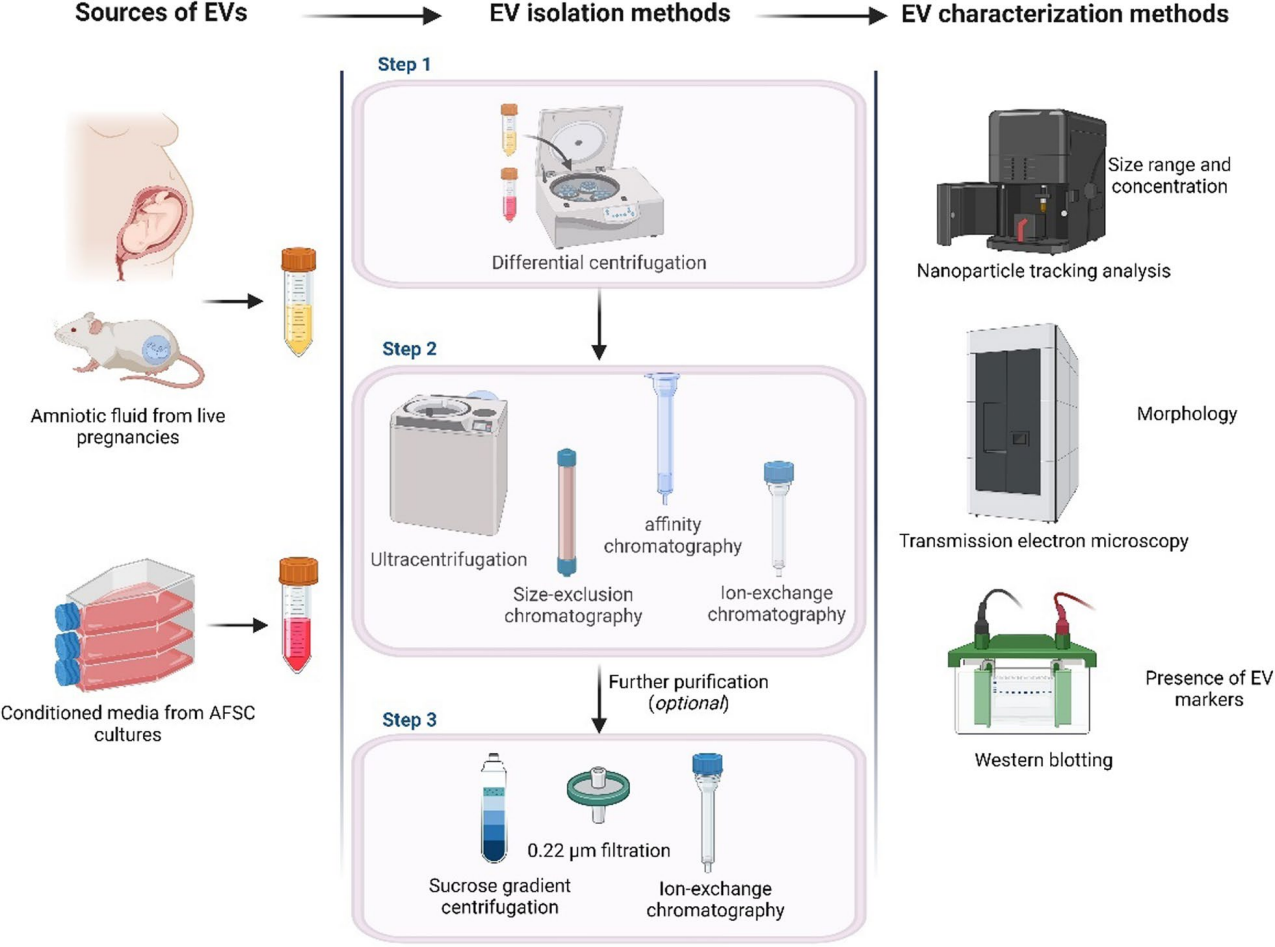
Commonly employed EV isolation and characterisation methods. Human/animal AF or conditioned media of AF stem cell/MSC cultures are first subjected to differential centrifugation to remove cellular debris. The supernatant is subjected to ultracentrifugation/size-exclusion chromatography/affinity chromatography or a combination of these methods. An optional further purification of the isolated EV population is achieved using density gradient centrifugation, filtration, or ion-exchange chromatography. Isolated EVs are characterised using nanoparticle tracking analysis for EV concentration and size range, transmission electron microscopy for EV morphology and Western blotting to analyse EV protein markers. Figure created with BioRender.com

The established guideline for characterising EVs and confirming their successful isolation is the Minimal Information for Studies of Extracellular Vesicles (MISEV2018) statement approved by the International Society for Extracellular Vesicles [66]. This characterization involves three main steps: (i) nanoparticle tracking analysis to confirm the size range and concentration of the isolated vesicles, (ii) transmission electron microscopy to visualise their morphology, and (iii) screening for standard EV enriched markers such as Alix, TSG-101 and tetraspanins CD63, CD81 and CD9 (Fig. 2). Only 23 (36%) of the included studies employed all three characterisation methods.

## Amniotic fluid EVs are abundant and immunologically active

Human AF appears to be a more concentrated source of EVs compared to other bio-fluids, with AF-EVs concentrations up to 41-times higher than maternal plasma [67]. AF-derived exosomes are also reportedly smaller (~ 100 nm) than EVs of other sources and contain standard EV markers [54]. The predominant fetal renal origin of these vesicles has been suggested by the presence of tetraspanin CD24, kidney marker aquaporin-2 [68] and CD133 [32]. Other identified proteins in AF-EVs include an obscure, lower molecular weight CA125 species [69], tubulin and heat shock proteins Hsp72 and Hsc73 [70]. These extracellularly released heat shock-related proteins are known as alarmins and are expressed under hypoxic, immune or inflammatory stress conditions [71].

AF-EVs are known for their immunomodulatory properties, which can suppress T-cell activation and pro-inflammatory cytokine release in-vitro [72]. AF-EVs may act as both pro- and anti-inflammasome activating agents, potentially priming the fetal immunity owing to the presence of bacterial DNA in these vesicles [73]. Moreover, AF-EVs triggered epithelial-to-mesenchymal transition and myofibroblast activation in stem cells [74]. These studies have revealed important biological properties of AF-EVs, suggesting their many roles and potential uses.

## AF stem/stromal cell-derived EVs are bioactive and have distinct ‘omic profiles

The AFSC-EV therapeutics is a rapidly growing field of research. One of the first studies exploring AFSC-EVs reported on their active immunoregulatory properties [75]. A recent comparative study confirmed a 25% higher EV yield from AF stem cells compared to human bone marrow-derived stem cells, making them preferable for clinical applications [76]. They contain a significant amount of the biologically active molecules of the secretome of AF stem cells. AFSC-EVs contain miRNA, but not mRNA, suggesting their role in directly or indirectly regulating existing signalling pathways of recipient cells rather than enforcing new ones [47].

Researchers have suggested that AFSC-EVs are metabolically independent entities [77]. Equivalently, EVs isolated from semen of multiple species (human, canine, equine, and bovine origin) produced ATP intrinsically through the glycolytic pathway [78, 79]. Presence of active metabolic enzymes, particularly glyoxalases and MG-H1, in AFSC-EVs cargo [61] adds up to this concept.

AF-EVs contain anti-inflammatory, immunomodulatory, and free radical scavenging properties [39]. These functions are manifested by stabilizing telomere lengths [80], increasing cell adhesion and migration, and regulating cytokine production under inflammatory conditions [81] in recipient cells. These findings indicate that AF-EVs may indirectly modulate the maternal immune system, potentially preventing fetal rejection by the mother’s body.

Selecting the appropriate source of AF stem cells based on desired therapeutic outcome is essential as neonatal and perinatal AFSC-EVs possess distinct proteomic and transcriptomic profiles [82]. Second trimester amniocentesis-derived immature AFSC-EVs displayed pro-vasculogenic, pro-regenerative, and anti-aging properties, while term pregnancy-derived AFSC-EVs exhibited pronounced immune-modulatory and anti-inflammatory characteristics. However, both types of AFSC-EVs had a rich microRNA signature containing regenerative paracrine factors [82].

## Amniotic fluid derived EVs as potential biomarkers

### Exosomal shuttle RNA and fetal development

The RNA cargo in exosomes is known as exosomal shuttle RNA (esRNA) [83]. esRNA within AF-EVs is protected by the lipid membrane from digestion by nucleases, making transcripts readily available for diagnostic or prognostic purposes [22]. A number of biomarker discovery studies basing AF-EV esRNA have been published for fetal conditions such as congenital hydronephrosis [34], congenital diaphragmatic hernia [84], fetal alcohol exposure, osteogenic differentiation [35], congenital heart defects [85] and ureteropelvic junction obstruction [86]. However, these studies are yet to be translated into clinically useful predictors of perinatal outcomes.

### AF-EVs and parturition

Labour is an inflammation driven process. Resident and infiltrating immune cells in reproductive tissue [87, 88] and free cytokines in AF are associated with labour, both term and preterm [89–91]. Preterm labour, intra-amniotic inflammation and infection, all result in differential packaging of cytokines in AF-EVs [33]. Placental alkaline phosphatase (PLAP)/CD63 ratio in AF-EVs has been suggested as a marker for preterm birth and preterm premature rupture of membranes [30]. Others have postulated that fetal lung-derived EVs in AF may have a role in parturition, as they induced senescence-associated secretory phenotype and proinflammatory molecules in human amniotic epithelial cells in term pregnancies [31]. Moreover, transcription regulator HIF1α contained in AF-EVs impacts comparatively shorter interval between amniocentesis and parturition [92].

### AF-EVs in obstetric complications

AF-EVs have been studied in a limited number of obstetric complications. Elevated CD105 (endoglin) in AF-EVs resembled augmented angiogenesis in preeclampsia [32]. Others studied AF-derived microparticles in disseminated intravascular coagulation and hypotension in amniotic fluid embolism [67]. These fetal-origin EVs [93] were predominantly from apoptotic events of epithelial and leukocytic cells [94]. Their cargo included procoagulant molecules such as phosphatidylserine and tissue factor [95], and extrinsic tenase complexes [96].

Congenital cytomegalovirus infection is a common infection worldwide and may result in a range of undesirable outcomes including fetal death [97]. Identification of the association between the fetal infection and the EV-borne pro-inflammatory cytokine profile [98], may be a step towards predictive biomarkers for severity of fetal infection.

While these studies have revealed potential AF-EV-borne biomarkers for obstetric complications, they are primarily discovery-phase reports that require to be clinically validated.

## Therapeutic applications of AF-EVs and AFSC-EVs

AF and AF cell-derived EVs gained substantial interest as a therapeutic in regenerative medicine. Biological activity of these EVs is dependent on the treatment dose, rather than the specific size or purity of the isolated EV populations [99]. As a cell-free product loaded with bioactive molecules, they contain many desirable properties. EVs have been shown to modulate inflammation [58, 100–102], curb oxidative stress [103] and augment wound healing [104, 105], ultimately leading to tissue regeneration. Moreover, as a natural cell-derived product, EVs present advantages such as biocompatibility and minimal toxicity for recipients. A summary of the preclinical and clinical therapeutic studies retrieved from our literature search is presented in Table 6.

**Table 6:** Potential therapeutic applications of AF-EVs and AF stem cell/MSC derived EVs for various organs/tissues

Disease condition	Therapeutics	Experiment design	Study conclusion
Lungs			
Bronchopulmonary dysplasia [39]	AF-EVs	Neonatal rat model	Treatment reduced pro-inflammatory cytokine production and free-radical quenching, conserving alveolar growth
Severe acute respiratory syndrome due to COVID-19 infection [41, 42, 122]	“Zofin” an FDA-approved AF-EVs therapeutic	Pilot clinical trials in severely ill COVID-19 patients	Treatment improved clinical status of participants and prevented disease progression
Fetal lung underdevelopment (pulmonary hypoplasia) [117]	AFSC-EVs	Fetal rabbit model of pulmonary hypoplasia, ex-vivo fetal rat lungs grown for 72 h	Treatment altered gene expression in hypoplastic lungs and restored branching morphogenesis and alveolarization, promoting tissue maturation and cellular homeostasis
Fetal pulmonary hypoplasia [65]	AFSC-EVs	Fetal rat pulmonary hypoplasia model	Treatment restored autophagy hypoplastic lungs by transferring EV-borne miRNA cluster miR-17∼92
Fetal pulmonary hypoplasia [116]	AFSC-EVs	Lung explants from fetal rat pulmonary hypoplasia model	Treatment rescued airspace density and branching morphogenesis promoting differentiation of lung cells during both canalicular and saccular stages of fetal lung development
Brain/neuroinflammation			
Neonatal hypoxic encephalopathy [124]	AF-EVs	Neonatal hypoxic mouse model	Treatment eased hypoxic encephalopathy and enhanced angiogenesis, improved performance of the spatial memory
Autism [125]	AF-EVs	Induced chick embryo autistic model	AF-EVs are effective drug delivery vehicles; successful unloading of sulforaphane resulted in gene expression regulation
Ischemic stroke [49]	AFSC-EVs	Ischemia/reperfusion in-vitro model	Treatment activated pro-survival and anti-apoptotic pathways
Alzheimer’s disease [50]	AFSC-EVs	Alzheimer’s disease neuron primary culture (murine)	Treatment reduced the progression of Amyloid-β-induced neuronal death and Alzheimer’s disease by improving neuron morphology and viability
Alzheimer’s disease [58]	AFSC-EVs	In-vitro neuroinflammation model	Treatment reduced neuroinflammation, significantly recovering cells from neurotoxicity
Neuromuscular junction integrity during muscle atrophy [103]	AFSC-EVs	Inducible in-vitro model of muscle atrophy	Treatment reduced disease progression, by protecting motor neurons from atrophic muscle cells-induced oxidative stress
Intestinal tissues			
Necrotizing enterocolitis [52]	AFSC-EVs	Inducible neonatal rat model	Treatment attenuated the bowel condition by activating Wnt/β-catenin signalling pathway
Necrotizing enterocolitis [51]	AFSC-EVs	Premature rat pup model	Treatment reduced the incidence and disease severity of experimental necrotizing enterocolitis
Necrotizing enterocolitis [100]	AFSC-EVs	Postnatal inducible mouse pup model	Treatment reduced intestinal injury and inflammation improving intestinal cell proliferation
Necrotizing enterocolitis [101]	AFSC-EVs	Postnatal inducible mouse pup model	Treatment reduced intestinal injury, NEC score, systemic and ileal inflammation, and NEC-associated brain injury
Inflammatory bowel disease [102]	AFSC-exosomes	Inducible in-vitro model of intestinal inflammation	Treatment reduced the severity of inflammation by downregulating inflammatory cytokines
Heart			
Cardiac muscle injury [47]	AFSC-EVs	Cardiotoxin injury mouse model	Treatment promoted tissue regeneration
Cardiac fibrosis [129]	AFSC-EVs	Induced-cardiac fibrosis in vitro model	Treatment improved angiogenesis
Cardiac injury [48]	AFSC-EVs	myocardial infarction rodent model	Treatment maintained the myocardial renewal with significant improvement of cardiac function
Ischemia–reperfusion injury [60]	AFSC-EVs	Non‑recovery ischaemia–reperfusion injury rat model	Treatment showed significant benefits in cardio-protection and angiogenesis
Myocardial infarction [64]	AFSC-EVs	Neonatal myocardial infarction mouse model	Developmentally immature AFSC-EVs are more effective in cardiomyocyte renewal and cell cycle re-entry
Skin			
Wound healing [104]	AFSC-EVs	Full-thickness skin-wounded rat model	Treatment accelerated the wound healing rate, enhancing regeneration of hair follicles, blood vessels and nerves. It also promoted cutaneous cell proliferation and collagen distribution
Wound healing [105]	AFSC-EVs	Full-thickness skin-wounded rat model	Treatment significantly attenuated the scar formation and fibrosis
Ovaries			
Ovarian failure due to chemotherapy [46]	AFSC-EVs	Mice subjected to chemotherapy	miR-146a and miR-10a in murine AFSC-EVs showed a dominant effect on reducing the apoptosis in ovarian cells
Ovarian failure due to chemotherapy [128]	AFSC-EVs	Inducible premature ovarian dysfunction rat model	Treatment restored total follicular counts, anti-Müllerian hormone levels, regular estrous cycles and conception; EV borne miRNA-21 acts by regulating PTEN and caspase 3 apoptotic pathways
Ovarian failure due to chemotherapy [59]	AFSC-EVs	Mice subjected to chemotherapy	AFSC-EV borne miR-369-3p down-regulated apoptosis of ovarian granulosa cells
Skeleton			
Osteoarthritis [56]	AFSC-EVs	Inducible osteoarthritis rat model	Treatment produced near complete restoration of cartilage (positively correlated to TGFβ content in EVs) and polarized macrophages into EV-treated knee joints
Osteoporosis [57]	AFSC-EVs	dexamethasone treated human pre-osteoblast cell line	Treatment maintained the precursor cell potential and viability of cells, delaying bone loss in steroid-related osteoporosis
Kidneys			
Alport Syndrome [127]	AFSC-EVs	Alport mice	Treatment reduced cellular damage, demonstrating glomerulus-targeted disease intervention
Testis			
Azoospermia [40]	AF-EVs	Non-obstructive azoospermia rat model	Treatment improved spermatogenesis and sperm quality, restoring testicular function in azoospermia rats
Organ damage			
Cystinosis [115]	AFSC-EVs	Ctns knockout mice	Treatment reduced lysosomal cystine accumulation in target cells

Amniotic fluid stem cell derived EVs were used in many pre-clinical studies and pilot clinical studies, resulting in encouraging findings. This table summarises the research findings based on the organ/pathology of interest in each study. *FDA* Food and Drug Administration

## Discussion

AF is an accessible human fetal sample with significant biological value. However, until recently, it has been under-explored in reproductive medicine compared to other sources such as maternal plasma and placental tissue. Keller and colleagues first reported the detection of EVs in human and murine AF in 2007 [68], but the field remained quiescent until the past 4 years. There is an increased interest in AF derived biologics since 2020, making up for 64% of studies in this review.

Researchers have debated the optimal methods for EV isolation and their purity assessment for the last decade [63]. The community achieved consensus with the publication of the Minimal Information for Studies of Extracellular Vesicles guidelines [66] regarding basic isolation and characterization of EVs. However, EVs are a heterogenous group and cannot be separated by biogenesis using existing methods [18]. Therefore, nomenclature of the vesicles is challenging and will remain a discussion for the foreseeable future. At present, large EVs or small EVs seem to be the appropriate terms to describe an EV population, based on the employed isolation methods. Our review shows the inconsistent terminology (Table 1) used in reproductive EV research.

Researchers seem to prefer ultracentrifugation over other methods for AF-EVs and AFSC-EVs isolation (Tables 3 and 5). However, specific details such as durations of spins and speed were lacking in several studies. Ultracentrifugation is considered the “gold standard” method for EV isolation due to its reliability and optimal yield [106, 107]. However, EV samples isolated using ultracentrifugation require further purification methods to achieve homogeneity. The use of other methods such as commercially available chromatography columns and polymeric precipitation were observed when sample sizes were too small for centrifugation. Many factors such as the source material and its volume, EV size range of interest and the downstream use of the isolated EVs can influence the isolation methods. Nonetheless, the choice of isolation method largely appeared to be at the discretion of individual research groups. A clear and globally accepted, robust set of guidelines for the methodologies for AF derived EVs would benefit this emerging research field.

The laborious nature of the differential centrifugation and ultracentrifugation procedures limits the scalability for EV production for clinical use [108]. Commercial products are attractive solutions but have not gained widespread acceptance as only 17% of studies in this review have utilized them. Methodological studies have compared the commercial EV isolation kits versus ultracentrifugation [55], and the use of both methods together in the same protocol [54] resulting in varying inferences. Regardless of these time and labour effective new commercial products, ultracentrifugation remains the preferred method for most researchers. Studies have presented EV concentrations using a range of units such as particles per gram of EV proteins, vesicles per millilitre of fluid (it is unclear if the fluid refers to AF or the EV suspension buffer) and EV proteins (µg) per millilitre. Adoption of a standard unit such as vesicle number per millilitre/gram of starting material (body fluid/tissue) or per million cells would help advance the field by allowing more direct comparisons of results and facilitating replication of studies.

EV isolation from conditioned media requires specific conditions. Use of serum-free culture media or EV-depleted FBS in the media is widely accepted, to avoid introducing exogenous EVs. Other components such as antibiotics, growth factors and supplements can also affect EV biogenesis and their cargo [66]. Confluence of cells, culture temperature, percentage CO_2_, O_2_ and incubation time before EV isolation may all alter EV yield, quality and their biomolecule content [109, 110].

Therefore, it is important all information is reported accurately in publications and lack thereof may result in lack of reproducibility. Many groups studied RNA cargo in EVs to develop predictive disease biomarkers. However, the effect of different EV and evRNA purification methods for downstream sequencing and profiling is not known [18]. Standardization of methodologies and terminology for publications is of central importance going forward. The compliance of experimental protocols with good manufacturing practice guidelines is highly commendable, which improves the quality of research and reproducibility across laboratories, facilitating smooth clinical translation.

Only one clinical application for AF-EVs has progressed to human clinical trials, no doubt accelerated by the urgency to develop novel therapies during the COVID-19 pandemic. Zofin, a human AF derivative enriched for EVs, is being evaluated in COVID-19 patients with severe acute respiratory syndrome in three separate studies, by the same group (NCT05228899, NCT04657406, NCT04384445). These clinical trials are still in progress, but pilot studies have proved safe use of AF-EVs with improved clinical outcomes.

The appeal of AF-EVs for COVID-19 treatment lies in their anti-inflammatory properties and their potential to curb the ‘cytokine storm’ of severe disease. Another clinical trial in Israel (NCT04747574) administered CD24-loaded EVs derived from HEK293 cells to COVID-19 patients, with encouraging outcomes [111]. Several other groups have also manifested the safety and feasibility of using acellular AF (not enriched for EVs) to treat COVID-19 patients in the clinic [112, 113]. Treatments for other inflammatory diseases also have shown the capacity of both AF-EVs and AFSC-EVs to reduce inflammation, restoring tissues or cells to their homeostatic state.

The number of clinical trials using AF-EVs or AFSC-EVs is currently minimal. However, clinical trials have used processed or unprocessed AF to treat chronic wounds (NCT04438174), osteoarthritis (NCT03074526, NCT02768155, NCT04886960), stenosing tenosynovitis (NCT03583151) and venous stasis ulcer (NCT04647240) among many others. The need for expertise, purpose-built instrument and laborious nature of isolating EVs may have delayed AF derived EV research reaching clinical translation.

Regenerative properties of AF-EVs and AFSC-EVs were used to treat necrotizing enterocolitis, premature ovarian failure and wound healing [99, 114]. Most studies demonstrated the desirable outcomes of these EV treatments in in-vitro and in-vivo models and some studies deciphered the underlying molecular mechanisms. In-depth understanding of the mechanisms will be beneficial in translating the findings to clinical applications. For example, AFSC-EVs treatment of cystinosis may have revealed a prospective targeted therapy for this rare disease, as the EVs were naturally loaded with cystinosin and reprogrammed the recipient mutant cells [115].

**Fig. 3:**
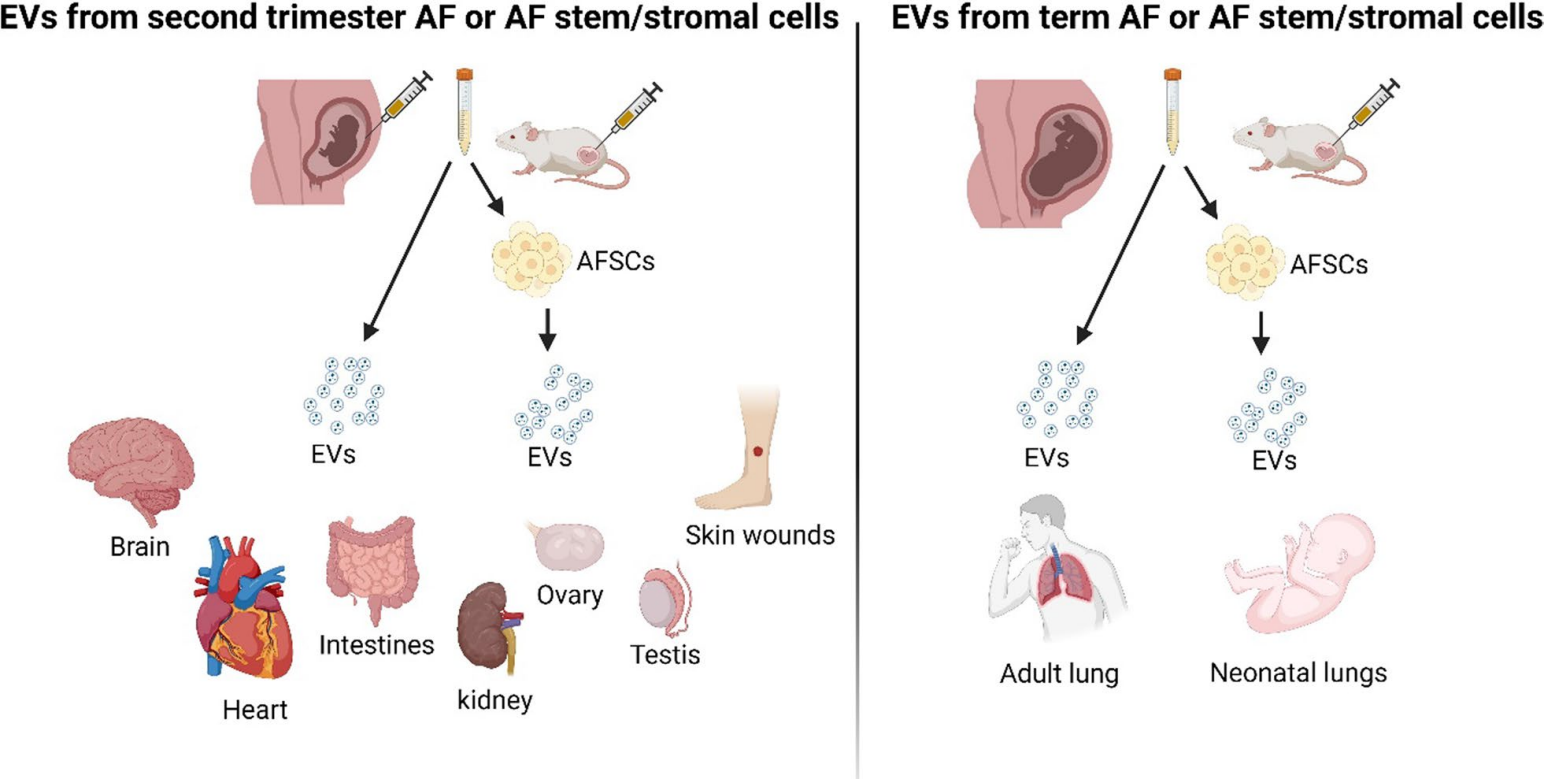
Gestation of amniotic fluid is matched with intended therapeutic use. The gestation at which the AF was collected was often matched to the therapeutic purpose of the research studies/clinical trials. For example, second trimester AF derived EVs were used when the regenerative properties of EVs were desired whereas third trimester AF derived EVs were preferred for lung function therapies. Researchers obtained second trimester AF from amniocentesis and third trimester AF from labour/Caesarean section at term. Figure created with BioRender.com

Stem cell-EV therapy has emerged as an attractive alternative to stem cell therapy, as it omits the challenges of unpredictable host rejection and poor efficacy. The shift in interest was promoted by research studies increasingly implying that the therapeutic effect of stem cells is mediated by the extracellular paracrine factors exerted via EVs [38]. Many research studies have demonstrated the successful utility of AFSC-EVs in pre-clinical models to treat different pathologies including necrotizing enterocolitis [51, 52, 100, 101], hypoplastic neonatal lungs [65, 116, 117] and wound healing [104, 105]. AF composition is dynamic and often represents the gestation-dependent development of fetal organs [118, 119]. Accordingly, the careful choice of gestation for AF collection according to the intended purpose of EVs was observed in these studies (Fig. 3). For example, for lung function-related therapies, AF obtained from elective Caesarean sections at term was used for EV or stem cell isolation, as fetal lungs rapidly develop close to parturition [120]. For other conditions, such as treating wound healing and necrotising enterocolitis, researchers used samples from second-trimester amniocentesis, where the AF is rich with factors implicated in tissue regeneration.

Our understanding of the biological difference between AF-EVs and AFSC-EVs is narrow and therefore there is currently no definitive evidence to propose biological superiority of one over the other. They conceivably are not bioequivalent and cannot be used inter-changeably. This is a grey area that has not been looked at yet. Researchers seem to be interested in EVs from both sources alike. Thirty-four (49%) articles included in this review used AF-EVs while 35 (51%) used AFSC-EVs. Since AFSC-EVs originate from one cell type, presumably they have minimal batch variations and more predictable biological properties compared to AF-EVs—both beneficial properties for clinical use. Therefore, a comprehensive comparison between AF-EVs and AFSC-EVs can benefit their applications.

If these EVs clear the hurdles to become therapeutics, AF collection and processing mechanisms will need to be increased and standardised. Additional research is needed to assess the inherent variation in AF samples from different donors and the suitability of singular or pooled samples for clinical applications. Despite the great excitement, there is a real risk that many studies of EVs as prognostic markers or therapies may be lost in the ‘valley of death’ between preclinical studies and clinical trials [121]. Therefore, further research, together with standardisation, may immensely progress the translation of these findings into clinical applications.

### Supplementary Information

**Additional file 1: Table S1.** List of included research studies.

## Data Availability

Data sharing not applicable to this article as no datasets were generated or analysed during the current study.

## References

[1] Underwood MA, Gilbert WM, Sherman MP (2005). Amniotic fluid: not just fetal urine anymore. J Perinatol.

[2] Wintour EM, Shandley L (1993). Effects of fetal fluid balance on amniotic fluid volume. Semin Perinatol.

[3] Moore TR (2010). Amniotic fluid dynamics reflect fetal and maternal health and disease. Obstet Gynecol.

[4] Sherer DM (2002). A review of amniotic fluid dynamics and the enigma of isolated oligohydramnios. Am J Perinatol.

[5] Beall MH, van den Wijngaard JP, van Gemert MJ, Ross MG (2007). Amniotic fluid water dynamics. Placenta.

[6] Shamsnajafabadi H, Soheili ZS (2022). Amniotic fluid characteristics and its application in stem cell therapy: a review. Int J Reprod Biomed.

[7] Ross MG, Nijland MJM (1998). Development of ingestive behavior. Am J Physiol Regul Integr Comp Physiol.

[8] Rabinowitz R, Peters MT, Vyas S, Campbell S, Nicolaides KH (1989). Measurement of fetal urine production in normal pregnancy by real-time ultrasonography. Am J Obstet Gynecol.

[9] Pierce J, Jacobson P, Benedetti E, Peterson E, Phibbs J, Preslar A (2016). Collection and characterization of amniotic fluid from scheduled C-section deliveries. Cell Tissue Bank.

[10] Tong XL, Wang L, Gao TB, Qin YG, Qi YQ, Xu YP (2009). Potential function of amniotic fluid in fetal development–-novel insights by comparing the composition of human amniotic fluid with umbilical cord and maternal serum at mid and late gestation. J Chin Med Assoc.

[11] Mao Y, Pierce J, Singh-Varma A, Boyer M, Kohn J, Reems J-A (2019). Processed human amniotic fluid retains its antibacterial activity. J Transl Med.

[12] Colombo M, Raposo G, Théry C (2014). Biogenesis, secretion, and intercellular interactions of exosomes and other extracellular vesicles. Annu Rev Cell Dev Biol.

[13] Meehan B, Rak J, Di Vizio D (2016). Oncosomes—large and small: what are they, where they came from?. J Extracell Vesicles.

[14] Pan BT, Teng K, Wu C, Adam M, Johnstone RM (1985). Electron microscopic evidence for externalization of the transferrin receptor in vesicular form in sheep reticulocytes. J Cell Biol.

[15] Harding C, Heuser J, Stahl P (1983). Receptor-mediated endocytosis of transferrin and recycling of the transferrin receptor in rat reticulocytes. J Cell Biol.

[16] Kang T, Atukorala I, Mathivanan S, Mathivanan S, Fonseka P, Nedeva C, Atukorala I (2021). Biogenesis of extracellular vesicles. New frontiers: extracellular vesicles.

[17] Abels ER, Breakefield XO (2016). Introduction to extracellular vesicles: biogenesis, RNA cargo selection, content, release, and uptake. Cell Mol Neurobiol.

[18] Witwer KW, Buzás EI, Bemis LT, Bora A, Lässer C, Lötvall J (2013). Standardization of sample collection, isolation and analysis methods in extracellular vesicle research. J Extracell Vesicles.

[19] Barry OP, Praticò D, Savani RC, FitzGerald GA (1998). Modulation of monocyte-endothelial cell interactions by platelet microparticles. J Clin Invest.

[20] Cossetti C, Iraci N, Mercer TR, Leonardi T, Alpi E, Drago D (2014). Extracellular vesicles from neural stem cells transfer IFN-γ via Ifngr1 to activate Stat1 signaling in target cells. Mol Cell.

[21] Kalluri R (2016). The biology and function of exosomes in cancer. J Clin Investig.

[22] Keller S, Ridinger J, Rupp A-K, Janssen JWG, Altevogt P (2011). Body fluid derived exosomes as a novel template for clinical diagnostics. J Transl Med.

[23] Sterzenbach U, Putz U, Low LH, Silke J, Tan SS, Howitt J (2017). Engineered exosomes as vehicles for biologically active proteins. Mol Ther.

[24] Du R, Wang C, Zhu L, Yang Y (2022). Extracellular vesicles as delivery vehicles for therapeutic nucleic acids in cancer gene therapy: progress and challenges. Pharmaceutics.

[25] Al-Dossary AA, Strehler EE, Martin-Deleon PA (2013). Expression and secretion of plasma membrane Ca2+-ATPase 4a (PMCA4a) during murine estrus: association with oviductal exosomes and uptake in sperm. PLoS ONE.

[26] Burns G, Brooks K, Wildung M, Navakanitworakul R, Christenson LK, Spencer TE (2014). Extracellular vesicles in luminal fluid of the ovine uterus. PLoS ONE.

[27] O'Neil EV, Burns GW, Spencer TE (2020). Extracellular vesicles: novel regulators of conceptus-uterine interactions?. Theriogenology.

[28] Nakamura K, Kusama K, Suda Y, Fujiwara H, Hori M, Imakawa K (2020). Emerging role of extracellular vesicles in embryo-maternal communication throughout implantation processes. Int J Mol Sci.

[29] Salomon C, Yee SW, Mitchell MD, Rice GE (2014). The possible role of extravillous trophoblast-derived exosomes on the uterine spiral arterial remodeling under both normal and pathological conditions. Biomed Res Int.

[30] Dixon CL, Sheller-Miller S, Saade GR, Fortunato SJ, Lai A, Palma C (2018). Amniotic fluid exosome proteomic profile exhibits unique pathways of term and preterm labor. Endocrinology.

[31] Wan S, Chen P, Gu M, Liu J, Zhou Q, Zhang F (2022). Fetal lung-derived exosomes in term labor amniotic fluid induce amniotic membrane senescence. Front Cell Dev Biol.

[32] Gebara N, Scheel J, Skovronova R, Grange C, Marozio L, Gupta S (2022). Single extracellular vesicle analysis in human amniotic fluid shows evidence of phenotype alterations in preeclampsia. J Extracell Vesicles.

[33] Bhatti G, Romero R, Rice GE, Fitzgerald W, Pacora P, Gomez-Lopez N (2020). Compartmentalized profiling of amniotic fluid cytokines in women with preterm labor. PLoS ONE.

[34] Xie J, Zhou Y, Gao W, Li Z, Xu Z, Zhou L (2017). The relationship between amniotic fluid miRNAs and congenital obstructive nephropathy. Am J Transl Res.

[35] Tavanasefat H, Li F, Koyano K, Gourtani BK, Marty V, Mulpuri Y (2020). Molecular consequences of fetal alcohol exposure on amniotic exosomal miRNAs with functional implications for stem cell potency and differentiation. PLoS ONE.

[36] Haney MJ, Klyachko NL, Zhao Y, Gupta R, Plotnikova EG, He Z (2015). Exosomes as drug delivery vehicles for Parkinson’s disease therapy. J Control Release.

[37] Costa A, Quarto R, Bollini S (2022). Small extracellular vesicles from human amniotic fluid samples as promising theranostics. Int J Mol Sci.

[38] Johnson J, Shojaee M, Mitchell Crow J, Khanabdali R (2021). From mesenchymal stromal cells to engineered extracellular vesicles: a new therapeutic paradigm. Front Cell Dev Biol.

[39] Bellio MA, Young KC, Milberg J, Santos I, Abdullah Z, Stewart D (2021). Amniotic fluid-derived extracellular vesicles: characterization and therapeutic efficacy in an experimental model of bronchopulmonary dysplasia. Cytotherapy.

[40] Mobarak H, Heidarpour M, Rahbarghazi R, Nouri M, Mahdipour M (2021). Amniotic fluid-derived exosomes improved spermatogenesis in a rat model of azoospermia. Life Sci.

[41] Mitrani MI, Bellio MA, Meglin A, Khan A, Xu X, Haskell G (2021). Treatment of a COVID-19 long hauler with an amniotic fluid-derived extracellular vesicle biologic. Respir Med Case Rep.

[42] Mitrani MI, Bellio MA, Sagel A, Saylor M, Kapp W, VanOsdol K (2021). Case report: administration of amniotic fluid-derived nanoparticles in three severely ill COVID-19 patients. Front Med (Lausanne).

[43] Viswanathan S, Shi Y, Galipeau J, Krampera M, Leblanc K, Martin I (2019). Mesenchymal stem versus stromal cells: International Society for Cell & Gene Therapy (ISCT®) mesenchymal stromal cell committee position statement on nomenclature. Cytotherapy.

[44] Robey P (2017). “Mesenchymal stem cells”: fact or fiction, and implications in their therapeutic use. F1000Res.

[45] Lindner U, Kramer J, Rohwedel J, Schlenke P (2010). Mesenchymal stem or stromal cells: toward a better understanding of their biology?. Transfus Med Hemother.

[46] Xiao GY, Cheng CC, Chiang YS, Cheng WT, Liu IH, Wu SC (2016). Exosomal miR-10a derived from amniotic fluid stem cells preserves ovarian follicles after chemotherapy. Sci Rep.

[47] Mellows B, Mitchell R, Antonioli M, Kretz O, Chambers D, Zeuner MT (2017). Protein and molecular characterization of a clinically compliant amniotic fluid stem cell-derived extracellular vesicle fraction capable of accelerating muscle regeneration through enhancement of angiogenesis. Stem Cells Dev.

[48] Balbi C, Lodder K, Costa A, Moimas S, Moccia F, van Herwaarden T (2019). Reactivating endogenous mechanisms of cardiac regeneration via paracrine boosting using the human amniotic fluid stem cell secretome. Int J Cardiol.

[49] Castelli V, Antonucci I, d'Angelo M, Tessitore A, Zelli V, Benedetti E (2021). Neuroprotective effects of human amniotic fluid stem cells-derived secretome in an ischemia/reperfusion model. Stem Cells Transl Med.

[50] Gatti M, Zavatti M, Beretti F, Giuliani D, Vandini E, Ottani A (2020). Oxidative stress in Alzheimer’s disease: in vitro therapeutic effect of amniotic fluid stem cells extracellular vesicles. Oxid Med Cell Longev.

[51] McCulloh CJ, Olson JK, Wang Y, Zhou Y, Tengberg NH, Deshpande S (2018). Treatment of experimental necrotizing enterocolitis with stem cell-derived exosomes. J Pediatr Surg.

[52] Li B, Lee C, O’Connell JS, Antounians L, Ganji N, Alganabi M (2020). Activation of Wnt signaling by amniotic fluid stem cell-derived extracellular vesicles attenuates intestinal injury in experimental necrotizing enterocolitis. Cell Death Dis.

[53] Ebert B, Rai AJ, Levy B (2019). Isolation and characterization of amniotic fluid-derived extracellular vesicles for biomarker discovery. Prenatal diagnosis.

[54] Sheller-Miller S, Menon R, Spada S, Galluzzi L (2020). Chapter ten—isolation and characterization of human amniotic fluid-derived exosomes. Methods in enzymology.

[55] Seyfizadeh N, Seyfizadeh N, Rahbarghazi R, Nourazarian A, Borzouisileh S, Palideh A (2019). Isolation and characterization of human amniotic fluid and SH-SY5Y/BE(2)-M17 cell derived exosomes. Acta Neurobiol Exp (Wars).

[56] Zavatti M, Beretti F, Casciaro F, Bertucci E, Maraldi T (2020). Comparison of the therapeutic effect of amniotic fluid stem cells and their exosomes on monoiodoacetate-induced animal model of osteoarthritis. BioFactors.

[57] Gatti M, Beretti F, Zavatti M, Bertucci E, Ribeiro Luz S, Palumbo C (2020). Amniotic fluid stem cell-derived extracellular vesicles counteract steroid-induced osteoporosis in vitro. Int J Mol Sci.

[58] Zavatti M, Gatti M, Beretti F, Palumbo C, Maraldi T (2022). Exosomes derived from human amniotic fluid mesenchymal stem cells preserve microglia and neuron cells from Aβ. Int J Mol Sci.

[59] Geng Z, Chen H, Zou G, Yuan L, Liu P, Li B (2022). Human amniotic fluid mesenchymal stem cell-derived exosomes inhibit apoptosis in ovarian granulosa cell via miR-369-3p/YAF2/PDCD5/p53 pathway. Oxid Med Cell Longev.

[60] Takov K, He Z, Johnston HE, Timms JF, Guillot PV, Yellon DM (2020). Small extracellular vesicles secreted from human amniotic fluid mesenchymal stromal cells possess cardioprotective and promigratory potential. Basic Res Cardiol.

[61] Romani R, Talesa VN, Antognelli C (2022). The glyoxalase system is a novel cargo of amniotic fluid stem-cell-derived extracellular vesicles. Antioxidants (Basel).

[62] Jia L, Li B, Fang C, Liang X, Xie Y, Sun X (2022). Extracellular vesicles of mesenchymal stem cells are more effectively accessed through polyethylene glycol-based precipitation than by ultracentrifugation. Stem Cells Int.

[63] Konoshenko MY, Lekchnov EA, Vlassov AV, Laktionov PP (2018). Isolation of extracellular vesicles: general methodologies and latest trends. Biomed Res Int.

[64] Costa A, Balbi C, Garbati P, Palamà MEF, Reverberi D, De Palma A (2022). Investigating the paracrine role of perinatal derivatives: human amniotic fluid stem cell-extracellular vesicles show promising transient potential for cardiomyocyte renewal. Front Bioeng Biotechnol.

[65] Khalaj K, Antounians L, Figueira RL, Post M, Zani A (2022). Autophagy is impaired in fetal hypoplastic lungs and rescued by administration of amniotic fluid stem cell extracellular vesicles. Am J Respir Crit Care Med.

[66] Théry C, Witwer KW, Aikawa E, Alcaraz MJ, Anderson JD, Andriantsitohaina R (2018). Minimal information for studies of extracellular vesicles 2018 (MISEV2018): a position statement of the International Society for Extracellular Vesicles and update of the MISEV2014 guidelines. J Extracell Vesicles.

[67] Uszyński W, Zekanowska E, Uszyński M, Zyliński A, Kuczyński J (2013). New observations on procoagulant properties of amniotic fluid: microparticles (MPs) and tissue factor-bearing MPs (MPs-TF), comparison with maternal blood plasma. Thromb Res.

[68] Keller S, Rupp C, Stoeck A, Runz S, Fogel M, Lugert S (2007). CD24 is a marker of exosomes secreted into urine and amniotic fluid. Kidney Int.

[69] Mitić N, Kosanović M, Milutinović B, Goč S, Mladenović D, Grubiša I (2018). Nano-sized CA125 antigen glycocamouflage: mucin—extracellular vesicles alliance to watch?. Arch Biochem Biophys.

[70] Asea A, Jean-Pierre C, Kaur P, Rao P, Linhares IM, Skupski D (2008). Heat shock protein-containing exosomes in mid-trimester amniotic fluids. J Reprod Immunol.

[71] Taha EA, Ono K, Eguchi T (2019). Roles of extracellular HSPs as biomarkers in immune surveillance and immune evasion. Int J Mol Sci.

[72] del Rivero T, Milberg J, Bennett C, Mitrani MI, Bellio MA (2022). Human amniotic fluid derived extracellular vesicles attenuate T cell immune response. Front Immunol.

[73] Nunzi E, Mezzasoma L, Bellezza I, Zelante T, Orvietani P, Coata G (2023). Microbiota-associated HAF-EVs regulate monocytes by triggering or inhibiting inflammasome activation. Int J Mol Sci.

[74] Liu N, Bowen CM, Shoja MM, Castro de Pereira KL, Dongur LP, Saad A (2022). Comparative analysis of co-cultured amniotic cell-conditioned media with cell-free amniotic fluid reveals differential effects on epithelial-mesenchymal transition and myofibroblast activation. Biomedicines.

[75] Romani R, Pirisinu I, Calvitti M, Pallotta MT, Gargaro M, Bistoni G (2015). Stem cells from human amniotic fluid exert immunoregulatory function via secreted indoleamine 2,3-dioxygenase1. J Cell Mol Med.

[76] Tracy SA, Ahmed A, Tigges JC, Ericsson M, Pal AK, Zurakowski D (2019). A comparison of clinically relevant sources of mesenchymal stem cell-derived exosomes: bone marrow and amniotic fluid. J Pediatr Surg.

[77] Mezzasoma L, Bellezza I, Orvietani P, Manni G, Gargaro M, Sagini K (2022). Amniotic fluid stem cell-derived extracellular vesicles are independent metabolic units capable of modulating inflammasome activation in THP-1 cells. FASEB J.

[78] Ronquist KG, Ek B, Morrell J, Stavreus-Evers A, Ström Holst B, Humblot P (2013). Prostasomes from four different species are able to produce extracellular adenosine triphosphate (ATP). Biochimica et Biophysica Acta (BBA) General Subjects.

[79] Ronquist KG, Sanchez C, Dubois L, Chioureas D, Fonseca P, Larsson A (2016). Energy-requiring uptake of prostasomes and PC3 cell-derived exosomes into non-malignant and malignant cells. J Extracell Vesicles.

[80] Radeghieri A, Savio G, Zendrini A, Di Noto G, Salvi A, Bergese P (2017). Cultured human amniocytes express hTERT, which is distributed between nucleus and cytoplasm and is secreted in extracellular vesicles. Biochem Biophys Res Commun.

[81] Bretz NP, Ridinger J, Rupp AK, Rimbach K, Keller S, Rupp C (2013). Body fluid exosomes promote secretion of inflammatory cytokines in monocytic cells via Toll-like receptor signaling. J Biol Chem.

[82] Costa A, Ceresa D, De Palma A, Rossi R, Turturo S, Santamaria S (2021). Comprehensive profiling of secretome formulations from fetal- and perinatal human amniotic fluid stem cells. Int J Mol Sci.

[83] Li J, Tian T, Zhou X (2019). The role of exosomal shuttle RNA (esRNA) in lymphoma. Crit Rev Oncol Hematol.

[84] Fabietti I, Nardi T, Favero C, Dioni L, Cantone L, Pergoli L (2021). Extracellular vesicles and their miRNA content in amniotic and tracheal fluids of fetuses with severe congenital diaphragmatic hernia undergoing fetal intervention. Cells.

[85] Yang H, Yang S, Shen H, Wu S, Ruan J, Lyu G (2021). Construction of the amniotic fluid-derived exosomal ceRNA network associated with ventricular septal defect. Genomics.

[86] Liu R, Zhang W, Luo M, Qin X, Yang F, Wei Q (2020). iTRAQ-based proteomics and in vitro experiments reveals essential roles of ACE and AP-N in the renin–angiotensin system-mediated congenital ureteropelvic junction obstruction. Exp Cell Res.

[87] Gomez-Lopez N, StLouis D, Lehr MA, Sanchez-Rodriguez EN, Arenas-Hernandez M (2014). Immune cells in term and preterm labor. Cell Mol Immunol.

[88] Bollopragada S, Youssef R, Jordan F, Greer I, Norman J, Nelson S (2009). Term labor is associated with a core inflammatory response in human fetal membranes, myometrium, and cervix. Am J Obstet Gynecol.

[89] Klebanoff M, Searle K (2006). The role of inflammation in preterm birth–focus on periodontitis. BJOG.

[90] Catov JM, Bodnar LM, Ness RB, Barron SJ, Roberts JM (2007). Inflammation and dyslipidemia related to risk of spontaneous preterm birth. Am J Epidemiol.

[91] Halgunset J, Johnsen H, Kjøllesdal AM, Qvigstad E, Espevik T, Austgulen R (1994). Cytokine levels in amniotic fluid and inflammatory changes in the placenta from normal deliveries at term. Eur J Obstet Gynecol Reprod Biol.

[92] Song JE, Park SJ, Lee KY, Lee WJ (2019). Amniotic fluid HIF1α and exosomal HIF1α in cervical insufficiency patients with physical examination-indicated cerclage. J Matern Fetal Neonatal Med.

[93] Butov KR, Karetnikova NA, Pershin DY, Trofimov DY, Panteleev MA (2022). Procoagulant activity in amniotic fluid is associated with fetal-derived extracellular vesicles. Curr Issues Mol Biol.

[94] Liu S, Wei L, Zhang Y, Xu M, Wang C, Zhou J (2014). Procoagulant activity and cellular origin of microparticles in human amniotic fluid. Thromb Res.

[95] Hell L, Wisgrill L, Ay C, Spittler A, Schwameis M, Jilma B (2017). Procoagulant extracellular vesicles in amniotic fluid. Transl Res.

[96] Hu Y, Repa A, Lisman T, Yerlikaya-Schatten G, Hau C, Pabinger I (2022). Extracellular vesicles from amniotic fluid, milk, saliva, and urine expose complexes of tissue factor and activated factor VII. J Thromb Haemost.

[97] Kenneson A, Cannon MJ (2007). Review and meta-analysis of the epidemiology of congenital cytomegalovirus (CMV) infection. Rev Med Virol.

[98] Bourgon N, Fitzgerald W, Aschard H, Magny J-F, Guilleminot T, Stirnemann J (2022). Cytokine profiling of amniotic fluid from congenital cytomegalovirus infection. Viruses.

[99] Antounians L, Tzanetakis A, Pellerito O, Catania VD, Sulistyo A, Montalva L (2019). The regenerative potential of amniotic fluid stem cell extracellular vesicles: lessons learned by comparing different isolation techniques. Sci Rep.

[100] O’Connell JS, Lee C, Farhat N, Antounians L, Zani A, Li B (2021). Administration of extracellular vesicles derived from human amniotic fluid stem cells: a new treatment for necrotizing enterocolitis. Pediatr Surg Int.

[101] Hu X, Zhang R, Liang H, An J, Yang Y, Huo J (2023). Comparison and investigation of exosomes from human amniotic fluid stem cells and human breast milk in alleviating neonatal necrotizing enterocolitis. Stem Cell Rev Rep.

[102] Katifelis H, Filidou E, Psaraki A, Yakoub F, Roubelakis MG, Tarapatzi G (2022). Amniotic fluid-derived mesenchymal stem/stromal cell-derived secretome and exosomes improve inflammation in human intestinal subepithelial myofibroblasts. Biomedicines.

[103] Gatti M, Dittlau KS, Beretti F, Yedigaryan L, Zavatti M, Cortelli P (2023). Human neuromuscular junction on a chip: impact of amniotic fluid stem cell extracellular vesicles on muscle atrophy and NMJ integrity. Int J Mol Sci.

[104] Zhang Y, Yan J, Liu Y, Chen Z, Li X, Tang L (2021). Human amniotic fluid stem cell-derived exosomes as a novel cell-free therapy for cutaneous regeneration. Front Cell Dev Biol.

[105] Wgealla M, Liang H, Chen R, Xie Y, Li F, Qin M (2022). Amniotic fluid derived stem cells promote skin regeneration and alleviate scar formation through exosomal miRNA-146a-5p via targeting CXCR4. J Cosmet Dermatol.

[106] Théry C, Amigorena S, Raposo G, Clayton A (2006). Isolation and characterization of exosomes from cell culture supernatants and biological fluids. Curr Protoc Cell Biol.

[107] Momen-Heravi F, Balaj L, Alian S, Mantel PY, Halleck AE, Trachtenberg AJ (2013). Current methods for the isolation of extracellular vesicles. Biol Chem.

[108] Colao IL, Corteling R, Bracewell D, Wall I (2018). Manufacturing Exosomes: a promising therapeutic platform. Trends Mol Med.

[109] Palviainen M, Saari H, Kärkkäinen O, Pekkinen J, Auriola S, Yliperttula M (2019). Metabolic signature of extracellular vesicles depends on the cell culture conditions. J Extracell Vesicles.

[110] Gudbergsson JM, Johnsen KB, Skov MN, Duroux M (2016). Systematic review of factors influencing extracellular vesicle yield from cell cultures. Cytotechnology.

[111] Shapira S, Ben Shimon M, Hay-Levi M, Shenberg G, Choshen G, Bannon L (2022). A novel platform for attenuating immune hyperactivity using EXO-CD24 in COVID-19 and beyond. EMBO Mol Med.

[112] Joseph ET, Jan P, Nathan H, Giavonni L, John DP, Alyssa M (2021). Safety and feasibility of using acellular sterile filtered amniotic fluid as a treatment for patients with COVID-19: protocol for a randomised, double-blinded, placebo-controlled clinical trial. BMJ Open.

[113] Selzman CH, Tonna JE, Pierce J, Vargas C, Skidmore C, Lewis G (2021). A pilot trial of human amniotic fluid for the treatment of COVID-19. BMC Res Notes.

[114] Beretti F, Zavatti M, Casciaro F, Comitini G, Franchi F, Barbieri V (2018). Amniotic fluid stem cell exosomes: therapeutic perspective. BioFactors.

[115] Iglesias DM, El-Kares R, Taranta A, Bellomo F, Emma F, Besouw M (2012). Stem cell microvesicles transfer cystinosin to human cystinotic cells and reduce cystine accumulation in vitro. PLoS ONE.

[116] Khalaj K, Figueira RL, Antounians L, Gandhi S, Wales M, Montalva L (2022). Treatment with amniotic fluid stem cell extracellular vesicles promotes fetal lung branching and cell differentiation at canalicular and saccular stages in experimental pulmonary hypoplasia secondary to congenital diaphragmatic hernia. Stem Cells Transl Med.

[117] Antounians L, Catania VD, Montalva L, Liu BD, Hou H, Chan C (2021). Fetal lung underdevelopment is rescued by administration of amniotic fluid stem cell extracellular vesicles in rodents. Sci Transl Med.

[118] Hui L, Slonim DK, Wick HC, Johnson KL, Bianchi DW (2012). The amniotic fluid transcriptome: a source of novel information about human fetal development. Obstet Gynecol.

[119] Tarca AL, Romero R, Pique-Regi R, Pacora P, Done B, Kacerovsky M (2020). Amniotic fluid cell-free transcriptome: a glimpse into fetal development and placental cellular dynamics during normal pregnancy. BMC Med Genomics.

[120] Mendelson CR, Montalbano AP, Gao L (2017). Fetal-to-maternal signaling in the timing of birth. J Steroid Biochem Mol Biol.

[121] Fernandez-Moure JS (2016). Lost in translation: the gap in scientific advancements and clinical application. Front Bioeng Biotechnol.

[122] Bellio MA, Bennett C, Arango A, Khan A, Xu X, Barrera C (2021). Proof-of-concept trial of an amniotic fluid-derived extracellular vesicle biologic for treating high risk patients with mild-to-moderate acute COVID-19 infection. Biomater Biosyst.

[123] Kosanović M, Milutinović B, Goč S, Mitić N, Janković M (2017). Ion-exchange chromatography purification of extracellular vesicles. Biotechniques.

[124] Li P, Lu X, Hu J, Dai M, Yan J, Tan H (2022). Human amniotic fluid derived-exosomes alleviate hypoxic encephalopathy by enhancing angiogenesis in neonatal mice after hypoxia. Neurosci Lett.

[125] Shahlaei M, Saeidifar M, Zamanian A (2022). Sustained release of sulforaphane by bioactive extracellular vesicles for neuroprotective effect on chick model. J Biomed Mater Res B Appl Biomater.

[126] Balbi C, Piccoli M, Barile L, Papait A, Armirotti A, Principi E (2017). First characterization of human amniotic fluid stem cell extracellular vesicles as a powerful paracrine tool endowed with regenerative potential. Stem Cells Transl Med.

[127] Sedrakyan S, Villani V, Da Sacco S, Tripuraneni N, Porta S, Achena A (2017). Amniotic fluid stem cell-derived vesicles protect from VEGF-induced endothelial damage. Sci Rep.

[128] Thabet E, Yusuf A, Abdelmonsif DA, Nabil I, Mourad G, Mehanna RA (2020). Extracellular vesicles miRNA-21: a potential therapeutic tool in premature ovarian dysfunction. Mol Hum Reprod.

[129] Hu J, Chen X, Li P, Lu X, Yan J, Tan H (2021). Exosomes derived from human amniotic fluid mesenchymal stem cells alleviate cardiac fibrosis via enhancing angiogenesis in vivo and in vitro. Cardiovasc Diagn Ther.

